# The effects of exercise training and type of exercise training on changes in bone mineral denstiy in Korean postmenopausal women: a systematic review

**DOI:** 10.20463/jenb.2016.09.20.3.2

**Published:** 2016-09-30

**Authors:** Jung Eun Kim, Hwasil Moon, Haeng Mi Jin

**Affiliations:** 1 Department of Nutrition Science, Purdue University, West Lafayette, IN USA; 2 Division of Kinesiology & Sport Studies, Ewha Womans University, Seoul Republic of Korea; 3 Department of Sport Rehabilitation, The Graduate School of Alternative Medicine, Kyonggi University, Seoul Republic of Korea

**Keywords:** Exercise, Bone mineral density, Postmenopausal women, Korean

## Abstract

**[Purpose]:**

To systematically review the effects of exercise training and the type of exercise training on changes in bone mineral density (BMD) in Korean postmenopausal women.

**[Methods]:**

Korean studies Information Service System (KISS) and PubMed were searched and 17 randomized control trials were selected. Changes in BMD of lumbar spine (LS), femur neck (FN), Ward’s triangle (WT), and trochanter (Tro) were chosen as major outcomes.

**[Results]:**

Exercise training increased BMD of LS, FN, WT, and Tro. According to the type of exercise training, combined exercise training (aerobic + resistance) showed improvements in BMD of LS, FN, WT, and Tro. However, aerobic exercise training alone and resistance exercise training alone showed inconsistent results.

**[Conclusion]:**

Exercise training can improve the BMD of LS, FN, WT, and Tro in Korean postmenopausal women. The type of exercise training may be a crucial factor for maintaining or improving bone health of this population.

## INTRODUCTION 

Osteoporosis is a major public health problem characterized by decreased bone mass and increased risk of bone fracture, resulting in reduced quality of life with substantial morbidity and mortality rates^1^. The prevalence of osteoporosis continues to increase progressively within an aging society, particularly in postmenopausal women^2^. International Osteoporosis Foundation has indicated that osteoporosis affects 200 million women worldwide and that 1 in 3 women over 50 years will experience osteoporotic fractures^1^. It has been reported that approximately 21% of women aged 50–84 years are classified as having osteoporosis in Europe and North America^1^. A recent study has also estimated that the prevalence of osteoporosis is 15.4% in US women aged 50 years and older using the National Health and Nutrition Examination Survey (NHANES) 2005-2010^3^. 

There is an emerging osteoporosis epidemic in most Asian countries^4^. Data from the nationwide survey of residents in Korea, the so-called Korea National Health and Nutrition Examination Survey (KNHANES), have shown that the prevalence of osteoporosis is 35.5% in Korean women aged 50 years or older^5^. The estimated annual national cost for osteoporosis care in Korea is more than US $1.4 billion and this cost continues to rise^6^. Therefore, management of osteoporosis for postmenopausal women is critical in Korea. 

Lifestyle modifications including exercise training has been recommended as an easy and cost-effective strategy to counter the loss of bone mass^7^. Although the specific mechanisms by which exercise training improves bone health remain unclear, it is widely accepted that exercise training stimulates the loading force on the bone, increases muscle mass, provides mechanical stress on the skeleton, and enhances osteoblast activity^8^^, ^^9^. Numerous studies have evaluated a range of exercise programs for their effects on bone mass, bone density, and bone strength in postmenopausal women. It has been suggested that resistance exercise training is primarily important to maintain or increase bone mass and bone density in this population^10^. Moreover, mounting evidence has indicated that a combination of aerobic and resistance exercise trainings can increase the levels of bone formation markers^11^. However, there is a paucity of data regarding the impact of aerobic exercise training on bone health^12^. 

Beneficial effects of exercise training on bone health, particularly on bone mineral density (BMD), in Korean postmenopausal women have been studied^13^^, ^^14^. However, limited studies have quantitatively assessed the effect of exercise training in this population. Furthermore, the impact of the type of exercise training on changes in BMD has not been systematically investigated. Therefore, the purpose of this systematic review was to evaluate the effects of exercise training and the type of exercise training on changes in BMD in Korean postmenopausal women. 

## METHOD 

The current systematic review followed the Preferred Reporting Items for Systematic Reviews and Meta-Analyses (PRISMA) guidelines^15^. The PICOS (population, intervention, comparison, outcome, and setting) criteria were used to define the research question as shown in Table 1. 

**Table 1:** The PICOS criteria used to define the research question.

Parameters	Description
Population	Postmenopausal women and/or older women whose mean age ≥ 65 years
Intervention	Groups who received exercise training
Comparison	Groups who did not receive exercise training
Outcome	Changes in bone mineral density of lumbar spine, femoral neck, Ward’s triangle, and trochanter
Setting	Randomized controlled trials
Research question	What is the effect of exercise training on changes in bone mineral density of lumbar spine, femur neck, Ward’s triangle, and trochanter in Korean postmenopausal women?

### Data source and inclusion criteria 

A computerized literature search was performed on April 15, 2015 by using Koreanstudies Information Service System (KISS, http://kiss.kstudy.com/) and PubMed (http://www.ncbi.nlm.nih.gov/pubmed). Inputted keywords included “Female AND Exercise AND bone mineral density” for KISS and “Korea[Mesh] AND Female[Mesh] AND Exercise[Mesh] AND Bone Density[Mesh]” for PubMed. Reference lists of the identified articles were reviewed to identify additional relevant articles. 

Studies were selected when they met all of the following inclusion criteria: 1) randomized control trials (RCT); 2) postmenopausal women and/or older women whose mean age ≥ 65 years; 3) receiving exercise training; and 4) using acceptable methods of BMD assessment such as dual energy X-ray absorptiometry (DXA). Primary outcomes for this study were changes in BMD of lumbar spine (LS), femoral neck (FN), Ward’s triangle (WT), and trochanter (Tro). 

### Literature search and data extraction 

**Figure 1.**
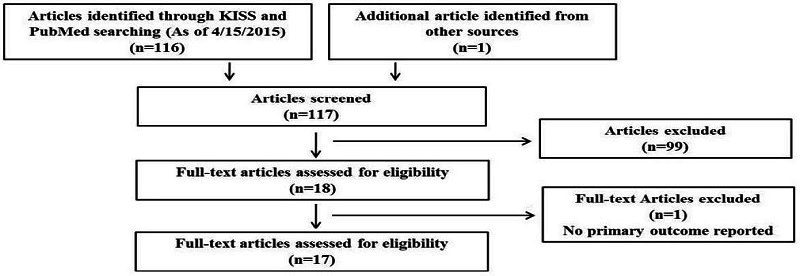
Flow chart of data extraction

KISS and PubMed searches yielded 116 articles. One article was identified as relevant from other reference lists. Literature search was conducted independently by the primary reviewer (JEK) and the secondary reviewer (HSM). Disagreements were resolved by consensus of investigators (JEK, HMJ, and HSM). Of the 117 articles, 99 were excluded due to the following reasons: study design was not a RCT; study subjects were premenopausal women or mean age < 65 years; exercise training was combined with other treatments including hormone replacement therapy or dietary intervention. Therefore, a total of 18 articles were included and fully assessed by JEK and the HSM independently. One article was excluded because it did not report primary outcomes. Finally, 17 articles were included for this systematic review (Figure 1). One article provided multiple treatments including dietary supplement and exercise training^16^.The group with dietary supplement treatment in this article was excluded since dietary supplement treatment did not meet our inclusion criteria. Four articles included two exercise groups^17^^, ^^18^ or three exercise groups^19^^, ^^20^ with different type of exercise training. Therefore, they were assigned into the groups with 2 and 3 different types of exercise training, respectively. 

The following data were extracted by the primary and secondary reviewers from each article using an electronic form: first author’s last name, publication year; population and sample size; details of intervention design; mean age; exercise type; intervention duration; method of outcome measures; pre- and post- intervention values; and changed value of outcome measures. 

### Quality assessment 

The quality of selected articles was assessed by using modified Cochrane risk of bias assessment tool^21^. Evaluated risk of bias included selection bias, performance bias, and detection bias. 

### Calculation 

Changes in BMD of LS, FN, WT, and Tro were manually calculated from raw pre- and post- intervention values reported in the articles. Percent change values of BMD were also manually calculated by dividing the change of BMD by pre-intervention value and multiplying the ratio by 100. 

## RESULTS 

### General characteristics of the studies 

Participants in 17 RCTs were either postmenopausal women, older women (mean age ≥ 65 years), or women who received hysterectomy. Interventions lasted between 8 to 48 weeks. Participants conducted exercise training 3-5 days per week. Eight^22^^-^^29^, five^18^^, ^^20^^, ^^30^^-^^32^, and two^16^^, ^^33^ articles provided combined (aerobic + resistance), aerobic, or resistance exercise training, respectively. Cho, 2003^17^ included both aerobic and combined exercise training groups. Ahn, 2002^19^ included both aerobic and resistance exercise training groups (Table 2). 

**Table 2:** Study characteristics

First author, year	Population (n) and mean age (years)	Study design/ Exercise type	Exercise protocol
Choi, 2011	EW (E: 16, C: 15) E: 69.7±2.5; C: 70.5±2.9	RCT/CE	5days/week, 48 weeks 10min WU; AE: 30~45min, HRR 40~60%; SE: 1RM x 50-70% SE: 1RM x 50-70%, 8-10 x 2sets and 10-15 x 1set; 10min CD
Kim, 2010	EW (E: 12, C: 12) E: 83.5±4.8; C: 83.4±4.0	RCT/RE	3days/week, 12 weeks 9min WU; RE: 40min, 1RM x 70%; 7min CD
Byeon, 2010	EW (E: 10, C: 10) E: 69.7±2.0; C: 68.5±3.7	RCT/CE	3days/week, 12 weeks 10min WU; AE: 20min, HRmax 40-75%; SE: 20min, 1RM x 8-12, 3sets; 10min CD
Kim, 2009	Women with Hysterectomy (E: 10, C: 10) E: 44.9±4.1; C: 46.1±6.1	RCT/CE	3days/week, 16 weeks AE: 20min, HRmax 55-70%; SE: 1RM x 40-50%, 10 x 3sets
Byeon, 2009	EW (E: 10, C: 10) E: 68.9±2.2; C: 68.5±3.7	RCT/CE	3days/week, 12 weeks 10min WU; AE: 20min, RPE 11-15; SE: 20min, RPE 11-15, 8-12 x 3sets; 10min CD
Park, 2008	EW (E: 25, C: 25) E: 68.3±3.6; C: 68.4±3.4	RCT/CE	3days/week, 48 weeks 9min WU; SE: 10min; AE: 23min, HRmax 65-75%; 18min CD
Park, 2004	EW (E: 13, C: 15) E: 70.1±2.4; C: 69.1±3.5	RCT/AE	3days/week, 12 weeks 15min WU; AE: 30min, HRR 60-80%; 15min CD
Sung, 2004	PW (E: 16, C: 16) E: 53.6±6.7; C: 56.3±4.4	RCT/RE	8 weeks 25min WU; RE: 1RM x 60-80%, 10-15 x 3sets; CD
Kim, 2004	PW (E: 10, C: 10) E: 48.9±4.1; C: 49.8±3.2	RCT/AE	4days/week, 16 weeks WU; AE: 40-45min, HRmax 60-85%;10min CD
Jee, 2003	EW (E: 17, C: 17) E: 75.8±3.9; C: 74.1±7.8	RCT/CE	5days/week, 48 weeks 15min WU; AE: 40-60min, VO2max 60-70%; SE: 1RM x 40-60%, 10-15 x 2sets; 20min CD
Choi, 2003	PW (E: 19, C: 19) E: 55.6±5.5; C: 57.4±4.3	RCT/CE	3days/week, 12 weeks 10min WU; AE: 35-40min, HRmax 60-80%; SE: 15min; 10min CD
Cho, 2003	PW (AE: 10, CE: 10, C: 10) AE: 43.2±1.9; CE: 44.1±2.0; C: 44.5±2.3	RCT/AE and CE	3days/week, 12 weeks AE: 30min: CE: AE: 20min; SE: 1RM x 60%, 12 x 3sets
Park, 2002	EW (E: 18, C: 17) E: 66.1±1.1; C: 67.0±0.9	RCT/CE	3days/week, 36 weeks 9min WU; AE: 23min, HRmax 65-75%; SE: 10min SE: 10min; 18min CD
Ahn, 2002	PW (AE1: 10, AE2: 10, SE: 10, C: 10) AE1: 53.5±4.1; AE2: 52.8±2.3; SE: 53.5±5.6; C: 52.5±4.6	RCT/AE and RE	4days/week, 48 weeks AE: 60min, HRmax 40-75% RE: 60min, 1RM x 40-75%, 7-10 x 3sets
Kim-1, 1999	PW (AE1: 7, AE2: 7, C: 8) AE1: 53.3±4.6; AE2: 56.0±3.1; C: 57.1±2.2	RCT/AE	4days/week, 12 weeks 5-10min WU; AE: 50min, HRmax 60%; 5-10min CD
Kim-2, 1999	PW (AE1: 8, AE2: 8, AE3: 8, C: 6) AE1: 52.8±4.6; AE2: 51.4±3.4; AE3: 53.6±4.2; C: 52.6±2.9	RCT/AE	5days/week, 12 weeks 5min WU; AE: 40min, HRmax 60-75%; 5min CD
Jung, 1996	PW (E: 8, C: 8) E: 51.6±3.3; C: 50.5±4.6	RCT/AE	4days/week, 16 weeks AE: 40-50min, VO2max 40-70%

Mean ± S.D.; E: exercise training; C: control; EW: elderly women; PW: postmenopausal women; RCT: randomized control trial; CE: combined exercise training; AE: aerobic exercise training; RE: resistance exercise training; WU: Warm up; CD: Cool down; RM: Repetition maximum, HRmax: Maximum heart rate; VO2max: Maximum oxygen uptake; RPE: rate perceived exertion.

### Quality of studies 

The qualities of selected articles are summarized in Table 3. Only one article^28^ indicated a low risk of randomization factor using computer-generated program for study randomization. The remaining studies did not clearly report the randomization method. Moreover, all articles did not provide details regarding allocation concealment, blinding of participants, study investigator, or blinding of outcome assessment. 

**Table 3:** Quality assessment

First author, year	Selection bias		Performance bias	Detection bias
	Randomization	Allocation concealment	Blinding of participants and study investigator	Blinding of outcome assessment
Choi, 2011	Unclear	Unclear	Unclear	Unclear
Kim, 2010	Unclear	Unclear	Unclear	Unclear
Byeon, 2010	Unclear	Unclear	Unclear	Unclear
Kim, 2009	Unclear	Unclear	Unclear	Unclear
Byeon, 2009	Unclear	Unclear	Unclear	Unclear
Park, 2008	Low Risk	Unclear	Unclear	Unclear
Park, 2004	Unclear	Unclear	Unclear	Unclear
Sung, 2004	Unclear	Unclear	Unclear	Unclear
Kim, 2004	Unclear	Unclear	Unclear	Unclear
Jee, 2003	Unclear	Unclear	Unclear	Unclear
Choi, 2003	Unclear	Unclear	Unclear	Unclear
Cho, 2003	Unclear	Unclear	Unclear	Unclear
Park, 2002	Unclear	Unclear	Unclear	Unclear
Ahn, 2002	Unclear	Unclear	Unclear	Unclear
Kim-1, 1999	Unclear	Unclear	Unclear	Unclear
Kim-2, 1999	Unclear	Unclear	Unclear	Unclear
Jung, 1996	Unclear	Unclear	Unclear	Unclear

### Reported findings 

The effects of exercise training on BMD are summarized in Table 4. All selected articles reported BMD change in LS after exercise training. In addition, BMD of FN, WT, and Tro were reported in 12^16^^-^^20^^, ^^22^^, ^^23^^, ^^27^^, ^^28^^, ^^30^^-^^32^, 11^16^^-^^20^^, ^^22^^, ^^23^^, ^^28^^, ^^30^^-^^32^, and 11^16^^-^^20^^, ^^22^^, ^^23^^, ^^28^^, ^^30^^-^^32^ articles, respectively. 

**Table 4:** Changes in bone mineral density of lumbar spine, femur neck, Ward’s triangle, and trochanter after exercise training

First author, year	Bone Assessment	Area	Δ (post-pre, g/cm^2^), Δ%
Choi, 2011	DXA	LS	E: +0.40, +4.7; C: -0.10, -1.1
Kim, 2010	DXA	LS	E: 0.002, +0.3; C: -0.001, -0.1
Byeon, 2010	DXA	LS	E: 0.016, +1.8; C: 0.008, +0.9
		FN	E: 0.011, +1.6; C: 0.005, +0.7
		WT	E: 0.016, +3.4; C: 0.009, +1.9
		Tro	E: 0.021, +3.7; C: 0.002, +0.4
Kim, 2009	DXA	LS	E: 0.02, +1.9; C: -0.01, -0.9
		FN	E: 0.03, +3.8; C: -0.01. -1.3
Byeon, 2009	DXA	LS	E: 0.071, +8.3; C: 0.008, +0.9
		FN	E: 0.016, +2.3; C: 0.005, +0.7
		WT	E: 0.013, +2.7; C: 0.009, +1.9
		Tro	E: 0.022, +3.8; C: 0.004, +0.7
Park, 2008	DXA	LS	E: 0.007, +0.7; C: -0.072, -7.5
		FN	E: 0.048, +5.9; C: -0.013, -1.7
		WT	E: 0.028, +4.4; C: -0.020, -3.4
		Tro	E: 0.030, +4.3; C: -0.032, -4.5
Park, 2004	DXA	LS	E: 0.120, +10.9; C: -0.010, -0.9
Sung, 2004	DXA	LS	E: -0.01, -1.1; C: 0.01, +1.1
		FN	E: 0.0, 0.0; C: 0.0, 0.0
		WT	E: 0.0, 0.0; C: -0.020, -3.2
		Tro	E: 0.0, 0.0; C: 0.0, 0.0
Kim, 2004	DXA	LS	E: 0.027, +2.6; C: -0.055, -5.4
		FN	E: 0.014, +1.7; C: -0.044, -5.3
		WT	E: 0.016, +2.3; C: -0.010, -1.4
		Tro	E: 0.035, +4.9; C: -0.024, -3.3
Park, 2004	DXA	LS	E: 0.120, +10.9; C: -0.010, -0.9
Jee, 2003	DXA	LS	E: 0.060, +7.1; C: 0.0, 0.0
Choi, 2003	DXA	LS	E: 0.0, 0.0; C: -0.021, -2.3
Cho, 2003	DXA	LS	AE: -0.010, -1.0; CE: 0.020, +2.0;C: -0.010, -1.0
		FN	AE: 0.010, +1.3; CE: 0.070, +8.5;C: -0.010, -1.2
		WT	AE: 0.010, +1.3; CE: 0.030, +3.9;C: -0.010, -1.3
		Tro	AE: 0.010, +1.4; CE: 0.020, +2.7;C: 0.010, +1.3
Park, 2002	DXA	LS	E: 0.03, +3.2; C: 0.0, 0.0
		FN	E: 0.05, +6.8; C: -0.03, -4.2
		WT	E: 0.01, +1.5; C: 0.01, +1.6
		Tro	E: 0.02, +2.9; C: 0.01, +1.6
Ahn, 2002	DXA	LS	AE1: 0.012, +1.1; AE2: 0.010, +0.9; SE: 0.019, +1.7; C: -0.024, -2.2
		FN	AE1: 0.008, +1.0; AE2: 0.003, +0.3; SE: 0.017, +2.1; C: -0.020, -2.4
		WT	AE1: 0.011, +1.5; AE2: -0.006, -0.9; SE: 0.015, +2.2; C: -0.012, -1.7
		Tro	AE1: -0.007, -1.0; AE2: 0.005, +0.7; SE: 0.011, +1.5; C: -0.020, -2.7
Kim-1, 1999	DXA	LS	AE1: 0.001, +0.1; AE2: 0.005, +0.5; C: 0.003,+0.3
		FN	AE1: 0.03, +3.6; AE2: -0.007,-0.9; C: -0.011,-1,4
		WT	AE1: 0.016, +2.4; AE2: -0.003,-0.5; C: -0.008,-1.2
		Tro	AE1: 0.001, +0.1; AE2: -0.005,-0.5; C: -0.007,-0.8
Kim-2, 1999	DXA	LS	AE1: 0.031, +3.1; AE2: 0.015, +1.5; AE3: 0.023, +2.1; C: 0.003,+0.3
		FN	AE1: 0.003, +0.4; AE2: 0.007, +0.8; AE3: 0.010, +1.2; C: -0.001, -0.1
		WT	AE1: 0.037, +5.3; AE2: 0.007, +1.0; AE3: 0.007, +0.9; C: 0.007, +0.1
		Tro	AE1: 0.011, +1.7; AE2: -0.003, -0.4; AE3: 0.048, 6.1; C: -0.008, -1.1
Jung, 1996	DXA	LS	E: 0.01, +1.0; C: -0.01,-1.3
		FN	E: 0.00,0.0; C: 0.01,+1.2
		WT	E: 0.00,0.0; C: -0.01,-1.3
		Tro	E: 0.00,0.0; C: -0.02,-2.7

DXA: dual energy X-ray absorptiometry; E: exercise; C: control; CE: combined exercise training; AE: aerobic exercise training; RE: resistance exercise training; LS: lumbar spine; FN: femoral neck; WS: Ward’s triangle; Tro: trochanter.

Exercise training resulted in greater positive change in BMD of LS. The average absolute and percent changes in BMD of LS were 0.02 g/cm^2^ (range: -0.01-0.12) and 2.3 % (range: -1.1-10.9), respectively, in the exercise group. In the control group without exercise training, the average absolute and percent changes in BMD of LS were -0.02 g/cm^2^ (-0.10-0.01) and -1.1 % (range: -7.5-1.1), respectively. In addition, the absolute changes in BMD of FN, WT, and Tro were 0.02 (range: -0.01-0.07), 0.01 (range: -0.01-0.04), and 0.01 g/cm^2^ (range: -0.01-0.05), respectively, in the exercise group and -0.01 (range: -0.04-0.01), -0.01 (range: -0.02-0.01), and -0.01 g/cm^2^ (range: -0.03-0.01), respectively, in the control group. 

**Figure 2.**
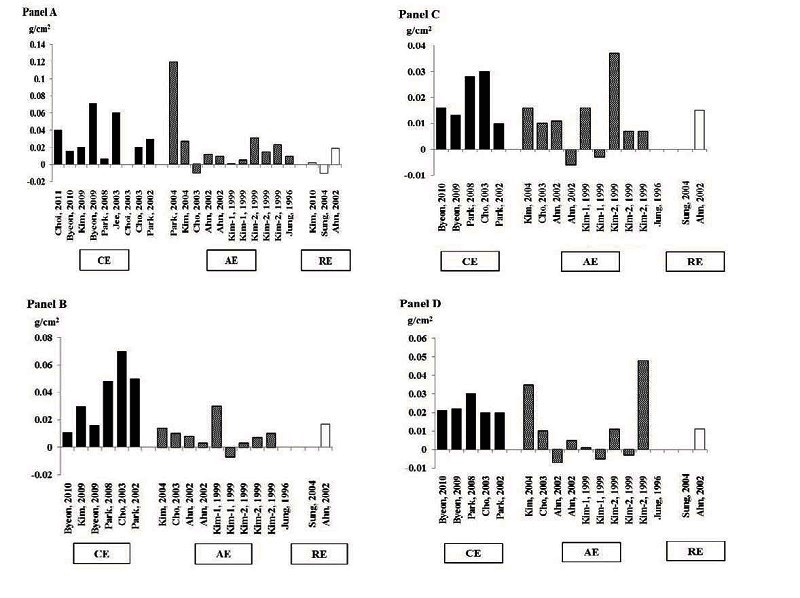
Changes in bone mineral density of lumbar spine (Panel A), femur neck (Panel B), Ward´s triangle (Panel C), and trochanter (Panel D) according to the type of exercise training. CE: combined exercise training; AE: aerobic exercise training; RE: resistance exercise training.

According to exercise type, the average absolute changes in BMD of LS, FN, WT, and Tro with combined exercise training were 0.03, 0.04, 0.02, and 0.02 g/cm^2^, respectively. The percent changes in BMD of LS, FN, WT, and Tro with combined exercise training were 3.3, 4.8, 3.2, and 3.4 %, respectively (Table 4 and Figure 2). Most of these articles indicated that both resistance and aerobic exercise trainings improved BMD. However, some articles reported that BMD was decreased after exercise trainings (Table 4 and Figure 2). 

## DISCUSSION 

Postmenopausal women present higher risk for bone mass loss with subsequent incidence of osteoporosis. Thus, understanding the role of preventative strategies to attenuate bone loss and inhibit the development of osteoporosis in this population is important^1^^, ^^7^. Although it is well known that exercise training is critical for the management of osteoporosis^1^, the ability of exercise training to counter the loss of bone mass has not been systematically evaluated in Korean postmenopausal women who are very likely to develop osteoporosis^4^^, ^^5^. Furthermore, there is a paucity of data on the impact of the type of exercise training on changes in BMD, an intermediate marker of bone health, in this population. Our finding suggests that exe rcise training can improve the BMD of LS, FN, WT, and Tro in Korean postmenopausal women. Although combined (aerobic + resistance) exercise training improves BMD of LS, FN, WT, and Tro, changes in BMD with aerobic or resistance exercise training alone are inconsistent. 

### Effect of exercise training on bone mineral density 

Bone is a dynamic tissue. It is very sensitive to mechanical stimuli such as exercise training that provides mechanical loading to bone^9^^, ^^10^. It has been well-established that exercise training may increase or maintain bone mass and decrease the risk of fracture in women during the aging process^9^. The current systematic review observed an overall increase in BMD of LS, FN, WT, and Tro with exercise trainings and a decrease in BMD without exercise training in Korean postmenopausal women. These results are consistent with previous systematic reviews and meta-analyses^34^^-^^37^. 

### Impact of length, intensity, and frequency of exercise training on bone mineral density 

Although positive impact of exercise training on BMD has been well-established, certain selected articles in this review have reported a minimal decrease or no changes in BMD after exercise training^17^^, ^^19^^, ^^20^^, ^^31^. Such results might be partially explained by the length of the exercise training. In general, bone remodeling requires a minimum of 4-6 months to 1-2 years^38^. The length of exercise training in these articles was ≤ 12 weeks, which is shorter than the time required for bone remodeling. Previous meta-analysis studies have reported beneficial impacts of exercise training on BMD by including articles that provided ≥ 24 weeks of exercise trainings^36^^, ^^37^. 

By contrast, another meta-analysis has reported no improvement in BMD of FN with ≥ 24 weeks of exercise training^39^ in postmenopausal women. The discrepancy in the results between our finding and previous work in FN might be due to differences in the intensity of exercise training. Most articles selected for previous research used lower intensity activities (primarily walking) as the exercise training protocol. A recent consensus statement from the National Institutes of Health Consensus Development Panel on Osteoporosis, Prevention, Diagnosis, and Therapy has suggested that higher intensity activities may develop higher peak bone mass and reduce the risk of fall in older adults^40^. 

Very limited human intervention studies have investigated the effect of exercise training frequency on bone. A recent study has reported that at least two sessions per week of exercise training might be crucial to observe the beneficial impact in postmenopausal and osteopenic women^41^. The United States Sports Academy has also recommended that three or more sessions per week using various types of exercises may increase BMD in older adults^42^. In the current systematic review, all selected articles provided three or more sessions per week which met the general recommendation. The optimum type of exercise training to improve bone health in postmenopausal women is not fully determined yet. Therefore, more studies are needed to evaluate the adequate length, intensity, and frequency of exercise training protocol in this population. 

### Effects of the type of exercise training on bone mineral density 

Although exercise training has been recommended for the prevention and management of postmenopausal bone loss, the most effective exercise type has not been well examined. In general, resistance exercise training has been highlighted as the most beneficial exercise type on bone health since a variety of muscular loadings are applied to the bone during resistance exercise training which generate stimuli and promote osteogenic response of the bone^43^. A previous systematic review has revealed that performing resistance exercise training 2-3 times per week during 1 year is able to maintain or increase the BMD of spine and hip in postmenopausal women^10^. A previous meta-analysis has also observed a positive effect of resistance exercise training in BMD of LS and femur in postmenopausal women^44^. Our systematic review included 3 articles that performed resistance exercise training. Consistent with previous findings, Ahn, 2002^19^ has reported improvements in BMD of LS, FN, WT, and Tro (1.5-2.2% increases) after 1 year of resistance exercise training. Kim, 2010^33^ has also reported a minimal increase (0.3%) after 12 weeks of resistance exercise training. By contrast, Sung et al., 2004^16^ have reported a minimal decrease or no changes in BMD of LS, FN, WT, and Tro after 8 weeks of resistance exercise training. The authors discussed that 8 weeks period might not be enough to observe the changes in BMD with resistance exercise training. 

Recently, mounting evidence has indicated that a combination of several types of exercise training may be more effective than any single type of exercise training for improving BMD in postmenopausal women^34^^, ^^45^. Particularly, a combination of resistance exercise training and weight-bearing aerobic exercise training including walking, running, or jumping is recommended since resistance exercise training provides muscular loading while weight-bearing aerobic exercise training provides additional mechanical loading to the bone above gravity. A recent systematic review has summarized that the majority of research studies with combined exercise training have shown improved BMD among postmenopausal women^34^. A previous meta-analysis to assess the effects of the types of exercise training has also revealed that a mixture of resistance and aerobic exercise trainings is effective in reducing postmenopausal bone loss at the hip and spine whereas other forms of exercise training are less effective in preserving BMD in this population^46^. Similar to previous findings, the articles that provided combined exercise training in the current review all reported improvements in BMD of LS, FN, WT, and Tro (0.7-8.5% increases). However, caution is warranted in the interpretation of positive impact of combined exercise training on BMD because methodology differences and reporting discrepancies still exist^34^^, ^^46^. 

There is a paucity of data regarding the impact of aerobic exercise training alone on bone health. Studies performed to determine the impact of aerobic exercise training on BMD in postmenopausal women have reported disparate results. One possible explanation for such disparate results is the application of weight-bearing versus non-weight-bearing aerobic exercise training in the exercise program. Many studies have observed positive effects of moderate to high impact weight-bearing aerobic exercise trainings on changes in BMD, including walking, jogging, stair climbing or mountain climbing, and jumping^34^^, ^^45^. However, particularly high-impact weight-bearing aerobic exercise trainings are not always suitable for older adults due to the presence of musculoskeletal impairment in this population^47^. Therefore, non-weight-bearing aerobic exercise training such as swimming has been recommended for this population. However, non-weight-bearing aerobic exercise training applied no or very low impact on bone. They had fewer osteogenic responses than weight-bearing aerobic exercise trainings in older adults^48^. The current systematic review also observed inconsistent results in BMD after aerobic exercise training among articles. Walking, jogging, aerobic dance, and mountain climbing (-1.0-10.9% increases) had greater improvements in BMD of LS, FN, WT, and Tro compared to swimming (-1.0-1.5% increases). Less favorable changes in bone status after aerobic exercise training have been reported compared to resistance^12^^, ^^49^ or combined^34^ exercise trainings. Consistent with previous studies, selected articles in the current review also reported greater increases of BMD with resistance and combined exercise trainings than aerobic exercise training alone. Ahn, 2002^19^ conducted aerobic or resistance exercise training and found that resistance exercise training (1.5-2.2% increases) provided more favorable changes in BMD of LS, FN, WT, and Tro compared to aerobic exercise training (-1.0-1.5% increases). Cho, 2003^17^ provided both aerobic and combined exercise training and found that combined exercise training (2.0-28.5% increases) had more improvements in BMD of LS, FN, WT, and Tro compared to aerobic exercise training alone (-1.0-1.4% increases). Although resistance and combined exercise trainings are more likely to improve BMD than aerobic exercise training in postmenopausal and older women, further studies are needed to evaluate the optimum type of exercise training in this population without causing difficulties to this population during exercise training. 

### Limitations and strengths 

The findings from the current systematic review were limited by various intervention design, intervention duration, and exercise training protocols. In addition, according to results from quality assessments, except Park, 2008^28^, all articles did not provide details regarding randomization, allocation concealment, blinding of participants and study investigator, and blinding of outcome assessment. Nonetheless, the current review systematically evaluated the impact of exercise training and the type of exercise training on BMD responses in Korean postmenopausal women who are at risk for osteoporosis. Moreover, LS, FN, WT, and Tro are the regions strongly attributable to osteoporosis^50^. Thus, the findings from this systematic review might be clinically useful for healthcare providers and Korean postmenopausal women. 

## CONCLUSION 

Participating in exercise training can improve the BMD of LS, FN, WT, and Tro in Korean postmenopausal women. The type of exercise training might be a crucial factor when researchers recommend an exercise training protocol to maintain or improve bone health in this population. 
